# Metagenomic Insights into the Taxonomic and Functional Features of Traditional Fermented Milk Products from Russia

**DOI:** 10.3390/microorganisms12010016

**Published:** 2023-12-21

**Authors:** Alexander G. Elcheninov, Kseniya S. Zayulina, Alexandra A. Klyukina, Mariia K. Kremneva, Ilya V. Kublanov, Tatiana V. Kochetkova

**Affiliations:** 1 Winogradsky Institute of Microbiology, Federal Research Center of Biotechnology of the Russian Academy of Sciences, Moscow 117312, Russia; zauylinakc@gmail.com (K.S.Z.); alexandra.a.popova@gmail.com (A.A.K.); kublanov.ilya@gmail.com (I.V.K.); kochetkova.tatiana.v@gmail.com (T.V.K.); 2 Faculty of Biology, Lomonosov Moscow State University, Moscow 119234, Russia; kremnevabio@gmail.com

**Keywords:** fermented milk products, metagenomics, lactic acid bacteria, secondary metabolites, antibiotic resistance, CAZymes

## Abstract

Fermented milk products (FMPs) contain probiotics that are live bacteria considered to be beneficial to human health due to the production of various bioactive molecules. In this study, nine artisanal FMPs (kefir, ayran, khurunga, shubat, two cottage cheeses, bryndza, khuruud and suluguni-like cheese) from different regions of Russia were characterized using metagenomics. A metagenomic sequencing of ayran, khurunga, shubat, khuruud and suluguni-like cheese was performed for the first time. The taxonomic profiling of metagenomic reads revealed that *Lactococcus* species, such as *Lc. lactis* and *Lc. cremoris* prevailed in khuruud, bryndza, one sample of cottage cheese and khurunga. The latter one together with suluguni-like cheese microbiome was dominated by bacteria, affiliated to *Lactobacillus helveticus* (32–35%). In addition, a high proportion of sequences belonging to the genera *Lactobacillus, Lactococcus* and *Streptococcus* but not classified at the species level were found in the suluguni-like cheese. *Lactobacillus delbrueckii*, as well as *Streptococcus thermophilus* constituted the majority in another cottage cheese, kefir and ayran metagenomes. The microbiome of shubat, produced from camel’s milk, was significantly distinctive, and *Lentilactobacillus kefiri*, *Lactobacillus kefiranofaciens* and *Bifidobacterium mongoliense* represented the dominant components (42, 7.4 and 5.6%, respectively). In total, 78 metagenome-assembled genomes with a completeness ≥ 50.2% and a contamination ≤ 8.5% were recovered: 61 genomes were assigned to the *Enterococcaceae*, *Lactobacillaceae* and *Streptococcaceae* families (the *Lactobacillales* order within *Firmicutes*), 4 to *Bifidobacteriaceae* (the *Actinobacteriota* phylum) and 2 to *Acetobacteraceae* (the *Proteobacteria* phylum). A metagenomic analysis revealed numerous genes, from 161 to 1301 in different products, encoding glycoside hydrolases and glycosyltransferases predicted to participate in lactose, alpha-glucans and peptidoglycan hydrolysis as well as exopolysaccharides synthesis. A large number of secondary metabolite biosynthetic gene clusters, such as lanthipeptides, unclassified bacteriocins, nonribosomal peptides and polyketide synthases were also detected. Finally, the genes involved in the synthesis of bioactive compounds like β-lactones, terpenes and furans, nontypical for fermented milk products, were also found. The metagenomes of kefir, ayran and shubat was shown to contain either no or a very low count of antibiotic resistance genes. Altogether, our results show that traditional indigenous fermented products are a promising source of novel probiotic bacteria with beneficial properties for medical and food industries.

## 1. Introduction

Fermented milk products (FMPs) are a “superfood” with a wide range of benefits for human health. Artisanal FMPs have been made following traditional recipes kept by local producers for centuries [1]. These products are naturally fermented either by a spontaneous process or by using specially selected starters (“back-sloping” method) resulting in FMPs varied in taste, texture, storability, health benefits, etc. As a result, traditional FMPs are inhabited by different microbial communities, characterized in particular by their beneficial properties to humans. With the development of the next generation sequencing (NGS) technologies, it has become possible to determine the community composition of such products quickly and quite reliably [2]. The metagenomic analysis, widely used to study environmental ecosystems, allows one to analyze the microbial ecology of food products in more detail and helps to reconsider their functionality. Metagenomic sequencing helps to identify the presence of species that have never been observed in FMPs before [3], as well as to monitor dynamic changes in the microbiome during the ripening (e.g., in cheese production) [4]. Moreover, metagenomics allows one to conduct a functional analysis of FMP microbiomes, i.e., to identify genes involved in the production of compounds affecting sensory and flavor characteristics of dairy products [5,6].

Traditions of consuming FMPs are widely distributed in Russia. Due to the multiethnicity of its population as well as other historical, cultural and geographical factors, the variety of artisanal FMPs prepared in different regions of Russia is enormous. Some of them are common in cultures and traditions of neighboring countries. For example, different brine cheeses such as suluguni and bryndza originated from the Caucasus region and Eastern Europe are also popular in many regions of Russia. The dairy products associated with the time of the rise of the Golden Horde, like koumiss, khurunga, khuruud, ayran and shubat are still prepared in Buryatia, Tuva and Altai regions. Some other products, such as kefir and ryazhenka, despite being produced by huge manufacturers nationwide and in other countries, are also produced locally by small households in the different regions of Russia. Occasional studies of microbial communities of artisanal local FMPs from Russia using NGS have previously been conducted, mainly in the regions closely located to China [7,8,9,10]. Recently, in order to study the microbial composition of traditional products, we performed a large-scale screening of 55 FMP samples belonging to 16 different product types from nine regions of Russia using 16S rRNA gene metabarcoding [11]. We found that all microbiomes were composed mainly of three dominant genera: *Lactobacillus*, *Lactococcus* and *Streptococcus*, and more than five nondominant genera grouped in *Firmicutes* (like *Lentilactobacillus*, *Leuconostoc*, *Lactiplantibacillus*, *Latilactobacillus*, *Enterococcus*) and *Proteobacteria* (*Acetobacter*) phyla of the bacteria domain. However, metabarcoding does not allow a reliable prediction of the functional features of the microbiomes inhabiting these products. Only recently, we published a metagenomics of traditional Caucasian cheeses’ microbiomes produced in Northern Ossetia-Alania [12]. According to the analysis of metagenome-assembled genomes (MAGs), it was revealed that the majority of dominating bacteria did not contain the genes of antibiotic resistance (ABR). At the same time, a great variety of genes of carbohydrate, protein and lipid metabolisms, as well as genes encoding the enzymes of useful biomolecules’ synthesis, such as gamma-aminobutyric acid or bacteriocins, were found.

Here, we report the results of metagenomic-sequencing-based taxonomic and functional analyses of various traditional FMPs, such as suluguni-like cheese, cottage cheese, bryndza, kefir, ayran, khurunga, khuruud and shubat for an in-depth characterization of the microbial constituents of these products and to provide additional knowledge about their probiotic potential, avoiding a culture-dependent routine.

## 2. Material and Methods

### 2.1. DNA Extraction and Metagenomic Sequencing

The details of sampling and DNA extraction were published earlier [11]. DNA libraries for shotgun metagenomic sequencing were prepared using KAPA HyperPlus kit (Kapa Biosysyems, Wilmington, MA, USA) according to manufacturer recommendations. The treatment included the enzymatic fragmentation of DNA (resulting in fragments with a length of 500–700 bp), end polishing and A-tailing, the ligation of specific adapters for sequencing (Nextera Mate Pair Library Prep Kit, Illumina, San Diego, CA, USA) and the amplification of the obtained libraries. Metagenomic sequencing was performed using Illumina NovaSeq 6000 platform (Illumina, San Diego, CA, USA).

### 2.2. Assembly of Metagenomes and MAGs Recovery

Raw reads from metagenomic sequencing were filtered using a trim tool (quality limit = 0.03, maximum ambiguous nucleotides = 2 and minimum length = 80) in CLC Genomic Workbench v.10 software (Qiagen, Hilden, Germany). Metagenomic assemblies were obtained using the metaSPAdes v3.15.5 with default settings [13]. Contigs with a length less than 500 bp were eliminated from the final assemblies. Binning to produce MAGs was performed using the metaWRAP v1.3.2 pipeline [14] with three tools: CONCOCT v.1.1.0 [15], MaxBin2 v.2.2.7 [16] and metaBAT2 v.2.12.1 [17]. Bin sets from different binning tools were consolidated using the Bin_refinement module of metaWRAP with the following parameters: a minimal completeness of 50% and a maximal contamination of 10%.

### 2.3. Taxonomic Assignment of Metagenomic Reads and Phylogenetic Analysis

The taxonomic assignment of metagenomic reads was conducted using the Kraken2 tool [18] and GTDB r89 database (the taxa names were adapted to r207 where it was possible).

The initial taxonomic classification of MAGs was performed using gtdb-tk v.2.1.1 [19] with the GTDB r207 database as reference. For the phylogenetic analysis based on the bac120 set of conserved proteins, protein sequences were identified and aligned using gtdb-tk v.2.1.1. A maximum-likelihood phylogenetic tree was constructed in RAxML v.8.12.2 [20] with the PROTGAMMAILG model of amino acid substitution and 1000 rapid bootstrap replications as support values.

### 2.4. Functional Analysis of Metagenomes

Gene calling and annotations of metagenomes were performed using NCBI Prokaryotic Genome Annotation Pipeline v.6.5 [21].

Carbohydrate-active enzyme (CAZyme) genes were searched in metagenomes using dbCAN v.4 [22] with HMMER tool [23].

Secondary metabolites biosynthesis clusters were identified by antiSMASH bacterial version 6.1 [24]. Antimicrobial resistance genes in metagenomes were determined using the ABRicate tool (https://github.com/tseemann/abricate accessed on 10 October 2023) and the CARD database [25] with the following thresholds: a minimal identity of 80% and a minimal coverage of 90%.

## 3. Results and Discussion

### 3.1. Taxonomic Composition of the Microbiomes

The metagenomes of nine artisanal FMPs sampled from different regions of Russia were sequenced with a high (>100×) coverage (Table 1). The sizes of assemblies varied from 12.88 Mbp (07KF) to 58.66 Mbp (03SU), while N50 values were 1005 bp (07KF)–18,726 bp (07BZ).

**Figure 1:**
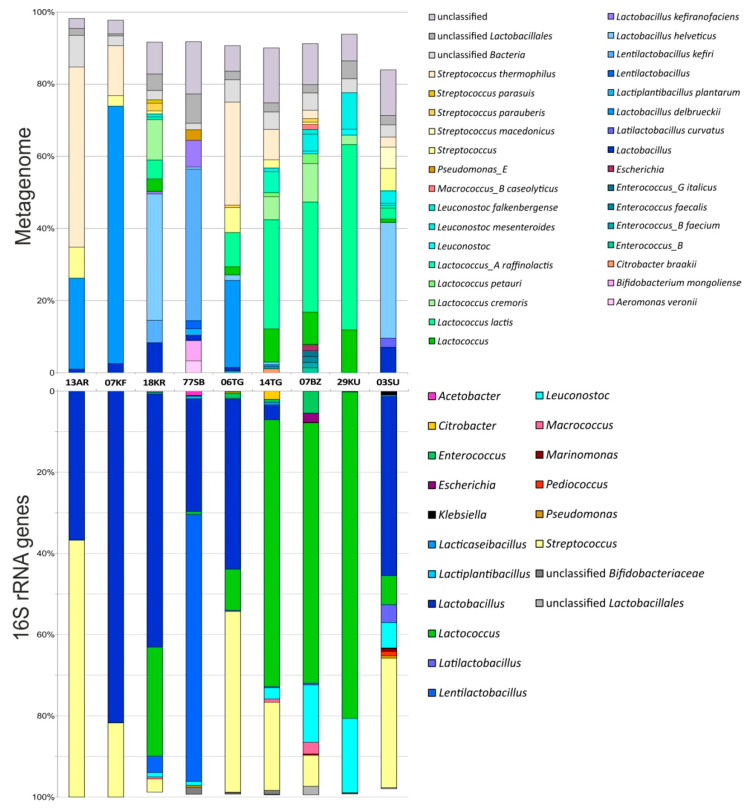
Microbiome composition of nine fermented milk products based on shotgun metagenomics (only species with ≥0.8% relative abundance) and 16S rRNA gene fragment profiling (revisualized data from our previous results [11]).

According to the taxonomic assignment of short reads, different representatives of lactic acid bacteria (LAB) were the most abundant between all products’ phylotypes, while the species-level composition depended on the product type (Figure 1; Table S1). It was found that *Lactobacillus delbrueckii* and *Streptococcus thermophilus* dominated in the microbiomes of both kefir (07KF) and ayran (13AR); however, the abundances of these species differed. The first one was more abundant in kefir (71.4% versus 25.2%), while the latter in ayran (50% versus 13.8%). Several species including *Lactobacillus helveticus* (35.1%), *Lactococcus cremoris* (11.2%), *Lactococcus lactis* (5.2%) and *Lentilactobacillus kefiri* (6.2%) were dominant taxa in the khurunga sample (18KR). At the same time, *Streptococcus parauberis*, *Streptococcus parasuis, Lactobacillus kefiranofaciens, Lactococcus raffinolactis* and *Leuconostoc mesenteroides* were present as minor species (0.7–2.1%). The shubat (77SB) microbial community was represented by *Lentilb. kefiri* (42%), *Lb. kefiranofaciens* (7.4%), *Bifidobacterium mongoliense* (5.6%), *Aeromonas veronii* (3.3%), *Lactiplantibacillus plantarum* (1.8%) and unclassified *Pseudomonas*_E species (2.9%). *Lb. helveticus* (0.6%) and *Lentilactobacillus farraginis* (0.5%) were minor species in the shubat microbiome. The microbiome of cottage cheese 06TG mainly consisted of *Lactobacillus* species, *Lb*. *delbrueckii* (24.1%) and *Lb*. *helveticus* (1.6%), *Streptococcus* species, *S*. *thermophilus* (28.6%) and *S*. *parauberis* (0.7%), as well as *Lc. lactis* (9.5%). Representatives of *Lacticaseibacillus paracasei* and *Enterococcus_B faecium* were minor components, 0.6% and 0.5% of the 06TG microbial community, respectively. The microbial community of another cottage cheese sample (14TG) differed significantly from the 06TG microbiome and contained *Lactococcus* species as the dominating microorganisms. Abundances of *Lc. lactis*, *Lc. cremoris*, *Lactococcus raffinolacis*, *Lactococcus petauri* and *Lactococcus garvieae* were 30.3%, 6.4%, 5.8%, 1.1% and 0.5%, respectively. Other components of the 14TG microbiome were *S. thermophilus* (8.4%), *Citrobacter braakii* (1.1%), *Lb. delbrueckii* (0.5%), *Lb. helveticus* (0.7%) and *Lcb. paracasei* (0.7%). Different species of *Lactococcus* genus also dominated in the microbiome of the 07BZ bryndza sample—*Lc*. *lactis* (30.5%), *Lc*. *cremoris* (10.6%) and *Lc*. *petauri* (2.7%). Minor components were represented by *Enterococcus faecalis* (1.7%), *Enterococcus_B faecium* (1.4%) and *Enterococcus_G italicus* (1.6%), two *Leuconostoc* species (the abundances of *Leu*. *mesenteroides* and *Leu*. *falkenbergense* were 4.7 and 1.2%, respectively), *S. thermophilus* (2.3%) and *Macrococcus caseolyticus* (1.4%). The microbiome of khuruud (29KU) mainly consisted of two *Lactococcus* species (*Lc. lactis* and *Lc. cremoris* with an abundance of 51.4% and 2.6%, respectively) and *Leu. mesenteroides* (10.1%). *Lb. helveticus* (32.1%) was the most abundant in the suluguni-like cheese (03SU), while *Streptococcus macedonicus* (5.9%) and *S*. *thermophilus* (2.8%), *Latilactobacillus curvatus* (2.6%), *Leu. mesenteroides* (3.5%), *Lc. lactis* (3.1%), *Lc. raffinolactis* (0.7%), *S. agalactiae* (0.7%), *Pediococcus pentosaceus* (0.7%) and *Lcb. paracasei* (0.6%) represented the minor components of the community. Moreover, a large proportion of reads affiliated to *Lactobacillus*, *Lactococcus* and *Streptococcus* genera but not classified at the species level was present there.

Generally, the whole metagenome-based analysis of the studied FMPs provided genus-level biodiversity data, similar to 16S rRNA gene metabarcoding [11]. The slight difference in microbial composition analyses obtained by these two approaches might be due to difficulties in the taxonomical assignment of some metagenomic reads because there is no clear threshold of assignment to the genus/species available for the 16S rRNA gene, for which a strong classification system exists [26].

### 3.2. Metagenome-Assembled Genomes

**Figure 2:**
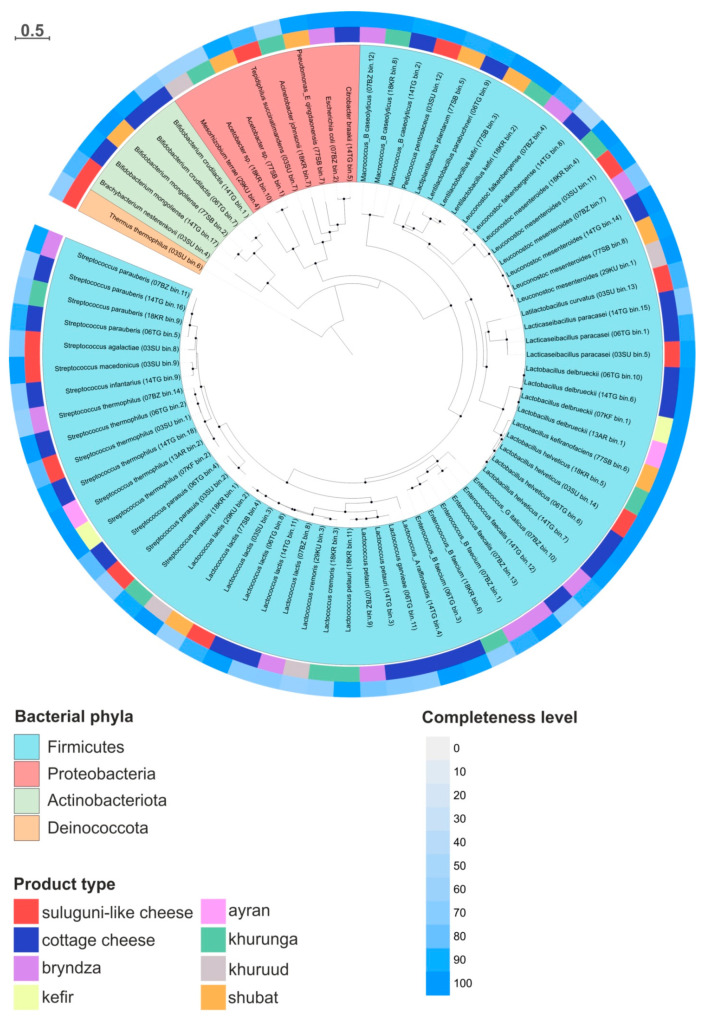
Phylogenetic tree based on the comparison of 120 conserved proteins and revealing the positions of metagenome-assembled genomes obtained from the metagenomes of nine fermented milk products.

In total, 78 MAGs with a completeness level above 50% and a contamination level below 10% were obtained (Table S2). The number of MAGs varied greatly and depended on the product type. Only two genomes were binned from the metagenomes of kefir (07KF) and ayran (13AR) samples while sixteen MAGs were obtained from the 14TG cottage cheese metagenome. The representatives of four bacterial phyla were identified, *Firmicutes* (synonym *Bacillota*), *Actinobacteriota*, *Proteobacteria* (synonym *Pseudomonadota*) and *Deinococcota* (Figure 2). The overwhelming majority of MAGs belonged to the *Lactobacillales* order—members of the *Lactobacillaceae* family (*P. pentosaceus*, *Lpb. plantarum*, *Lentilactobacillus parabuchneri*, *Lentilb. kefiri*, *Leu. falkenbergense*, *Leu. mesenteroides*, *Latilb. curvatus*, *Lcb. paracasei*, *Lb. delbrueckii*, *Lb. kefiranofaciens* and *Lb. helveticus*); representatives of the *Streptococcaceae* family (*Enterococcus_G italicus*, *Ec. faecalis*, *Enterococcus_B faecium*, *Lactococcus_A raffinolactis*, *Lc. garvieae*, *Lc. petauri*, *Lc. cremoris*, *Lc. lactis*, *S. parasuis*, *S. thermophilus*, *S. infantarius*, *S. macedonicus*, *S. agalactiae* and *S. parauberis*). Some species were cosmopolitan components, such as *S. thermophilus*, *Leu. mesenteroides* and *Lc. lactis*; they were presented in six studied FMPs, while other species were found only in a single product. *S. agalactiae*, *S. macedonicus*, *P. pentosaceus* and *Latilb. curvatus* were identified only in the metagenome of suluguni-like cheese (03SU), *S. infantarius* and *Lc. Raffinolactis* in the metagenome of cottage cheese 14TG, *Lc. garvieae* in the metagenome of cottage cheese 06TG, *Ec. italicus* in the 07BZ bryndza metagenome and *Lb. kefiranofaciens* in the metagenome of 77SB shubat.

The bacteria of *Lactobacillaceae* and *Streptococcaceae* families are responsible for lactic acid fermentation, the key step of FMP preparation. They can be divided into homo-, hetero-, or facultative fermentative bacteria. Homolactic fermentative LAB found in studied microbiomes included *Lc. lactis*, *S. thermophilus*, *Lb. delbrueckii*, *Lb. helveticus* and members of the *Enterococcus* genus [27]. Heterolactic fermentative LAB, capable of producing lactate, ethanol and CO_2_, included *Leuconostoc* species and several lactobacilli, for example *Latilactobacillus curvatus*. Microorganisms from the *Lactobacillaceae* family, like *Lb. helveticus*, *Lb. delbrueckii*, *Lpb. plantarum* and *Lcb. paracasei* dominated in some dairy products analyzed here and are also frequently detected in cheeses [5,10,12,28,29], yogurts, kefir and other fermented dairy products [8,30,31,32]. They appear to be one of the crucial LAB in milk fermentation due to their high potential for producing important metabolites and for improving the quality characteristics of the fermented products, for example, synthesizing thickening hydrocolloids (exopolysaccharides, EPS) or lipolysis-derivative aroma compounds [33,34]. Together with the members of the *Lactobacillaceae* family, *Lactococcus* representatives play an important role in milk fermentation. MAGs belonging to *Lc. lactis* were identified in creamy and solid dairy products and shubat while *Lc. cremoris* MAGs were only found in khurunga and khuruud. These species are typical components of starter cultures in the production of a wide range of FMPs, where they contribute to food preservation, flavor and texture formation [31,35,36]. Members of the *Leu. mesenteroides* accounted for significant quantities in cheese samples, but they were also detected in khurunga and shubat. It is well known that these bacteria are typical additional component of fermented food microbiomes (especially in cheeses) where they are responsible for specific flavors produced in the products, as well as their texture formation through the EPS synthesis [37,38,39]. Representatives of the *Streptococcus* genus are producers of large amounts of lactic acid, EPS and flavor compounds during fermentation processes. Certain species, like *S. thermophilus*, are considered as the second most important industrial dairy starter after lactococcii [40]. In addition to *S. thermophilus*, MAGs belonging to *S. macedonicus*, which also has important biotechnological properties similar to those of *S. thermophilus* [41], was detected in suluguni-like cheese.

Together with the canonical LAB listed above, *Macrococcus_B caseolyticus* (*Staphylococcales* order within the *Firmictutes* phylum) was present in some studied metagenomes (bryndza, cottage cheese (14TG only) and khurunga). Earlier it was detected in some kind of cheeses [12,42] and other fermented food [43,44] and can potentially enhance the release of flavor compounds in FMPs due to a high esterase activity [42]. Among *Proteobacteria* (currently *Pseudomonadota*), MAGs affiliated to novel species of the *Acetobacter* genus were only detected in khurunga and shubat samples. Members of the genus *Acetobacter* are often found in “kefir grains” [38,45] and, together with yeasts, produce CO_2_, alcohol and acetate, resulting in fizzing and a sour taste in kefir. Among other minor species found in the analyzed samples, e.g., suluguni-like cheese, namely thermophilic bacteria, which may result from environmental contamination, *Tepidiphilus succinatimandens* has never been reported in dairy foods. MAG belonging to *Mesorhizobium terrae* can also be attributed to contamination from the environment [46]; however, *Mesorhizobium* was detected in alcoholic beverages [47]. The presence of *Thermus thermophilus*, thermophilic bacterium belonging to the *Deinococcota* phylum, was not surprising, as this bacterium is sometimes found in pasteurized milk [48] or fermented milk products [49]. The preparation of suluguni-like cheese involves heating and melting the cheese mass at a temperature similar to the growth optimum of this bacterium. It is not a pathogenic microorganism, and its presence should not affect the safety of the product use; however, it can mediate a pink-defect formation in cheeses [50]. Thus, monitoring its presence is an important part of the cheese-making process.

*Actinobacteriota* phylum was represented by two genera, *Bifidobacterium* (*B. crudilactis* and *B. mongoliense*) and *Brachybacterium*. Both *Bifidobacterium* species were found in the 14TG cottage cheese, only *B. mongoliense* in shubat and only *B. crudilactis* in the 06TG cottage cheese. The presence of bifidobacteria in these products has not been shown before [51]. Many *Bifidobacterium* strains, along with LAB affiliated to *Firmicutes* phylum, have a “generally recognized as safe” (GRAS) status [52]. They are an important group of human gut microbiota since they have a number of beneficial probiotic effects on the host health, including the production of L(+) lactate isomers that are metabolized by human enzymes more easily. For this reason, bacteria of this group are often added to infant dairy [53]. However, most bifidobacteria cannot sustain the presence of oxygen, which makes their industrial application problematic. Nevertheless, some strains of *B. mongoliense* and *B. crudilactis* can tolerate the presence of oxygen and a low pH, providing the potential use of these species as probiotics [54]. A MAG affiliated to *Brachybacterium nesterenkovii* was identified in the metagenome of suluguni-like cheese (03SU). It is worth noting that *Brachybacterium* members were detected in various fermented foods including FMPs [55,56], but nothing is currently known about the beneficial properties of these bacteria.

It should be noted that some species were detected by sequencing read annotation (see Section 3.1) and/or MAGs recovery belonging to Risk Group 2 according to TRBA466 (https://lpsn.dsmz.de accessed on 13 December 2023). But the abundances of such species were very low in all products: in most FMPs, there were no observed “unwanted” reads/MAGs, or their presence was below 1%. In one case, the number of such bacteria was slightly higher—representatives of *Pseudomonas* and *Aeromonas* genera amounted to 2.9% and 3.3% in shubat, respectively. Some of the microorganisms from Risk Group 2, such as *Enterococcus_B faecium* (0.5, 0.6 and 1.4% of reads in cottage cheeses 06TG, 14TG and bryndza 07BZ, respectively; also, MAG was obtained for khurunga) and *Enterococcus faecalis* (1.7% of reads in bryndza; MAGs were recovered from the metagenomes of bryndza and cottage cheese 14TG) are normal symbionts of the human gut, but some species are capable of causing clinically important diseases [57]. However, they are often present in raw milk and can be normal components of starter culture during milk fermentation [58,59]. These species may even serve as a base for probiotic preparations [60], due to their activity against food spoilage bacteria and food-borne pathogens, cholesterol lowering ability, as well as EPS production [61]. Other unwanted species, like *Lc. Garvieae,* were identified in cottage cheeses (06TG and 14TG). Although this bacterium was initially isolated from cattle with mastitis, representatives of this species were shown to have probiotic properties [62] and to be a common component of some FMPs [63]. For other species detected in our analysis, like *S. infantarius*, *S. parasuis,* both the ability to cause human infections and to participate in milk fermentation have been shown [64,65,66]. As for the *Proteobacteria* representatives in some FMPs, such as *Citrobacter braakii* and *Escherichia coli*, their presence in food is unacceptable, due to their ability to produce significant quantities of unpleasant bioamines, like putrescine, histamine, spermine and spermidine [67]. A single MAG belonging to *Acinetobacter johnsonii* and reads assigned to *Aeromonas veronii* were detected in khurunga and shubat, respectively. It is worth keeping a close check on the presence of these species in food because it can cause serious diseases [68,69]. As for *Pseudomonas qingdaonensis* detected in the shubat sample, this aflatoxin-degrading bacterium often inhabits rhizosphere and soils [70], so in our case, it was probably a contaminant culture. Thereby, organisms from Risk Group 2 possibly enriched from the improper preparation of the product may be unwanted in foods and significantly reduce FMPs’ safety. Nevertheless, such organisms may also represent functional components of FMPs. In this case, it must be ensured that these strains are nonpathogenic, using not only in-depth functional genomics of pure cultures but also laboratory investigations.

### 3.3. Functional Analysis of Metagenomes

#### 3.3.1. Carbohydrate-Active Enzymes

**Figure 3:**
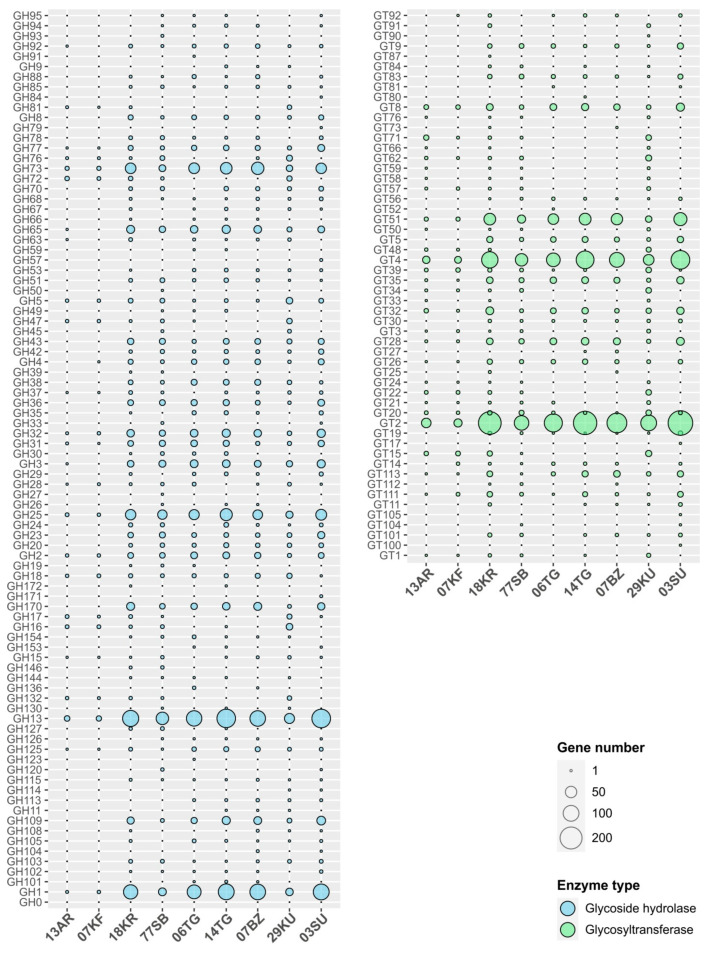
Glycoside hydrolases and glycosyltransferases encoded in the metagenomes of the studied fermented milk products: AR—ayran, KF—kefir, KR—khurunga, TG—cottage cheese, BZ—bryndza, KU—khuruud, SU—suluguni-like cheese.

Proteins, carbohydrates and other organic compounds present in milk participate in the microbiota formation of the fermented product. In turn, the microbial community inhabiting such products can produce enzymes or carbohydrates-containing compounds that improve fermentability, inhibit pathogenic bacteria, influence organoleptic properties, control sugar metabolism and so on [71]. CAZymes [72] are one of the most numerous and important groups of enzymes in FMP microbiomes. This is true for all known types of CAZymes that include glycoside hydrolases (GH), glycosyltransferases (GT), polysaccharide lyases (PL), carbohydrate esterase (CE), auxiliary activities/carbohydrate oxygenase (AA) and carbohydrate-binding modules (CBM). The total number of the genes encoding CAZymes in the analyzed metagenomes varied from 180 in the kefir metagenome (07KF) to 1457 in the suluguni-like cheese (03SU) metagenome (Table S3), that is, 1.1% and 1.8% of all protein-coding sequences in these metagenomes. GHs and GTs were the most numerous enzymes counting 59–653 and 102–648 genes, respectively, while other enzyme types were less represented—0–8 PL genes, 4–99 CE genes, 12–49 AA genes and 4–23 genes encoding proteins containing only CBMs (Figure 3). The predominance of glycosidases and glycosyltransferases over other CAZymes was also observed in other studies of FMP metagenomes [73] allowing us to suggest a crucial role of these enzymes in the FMP preparation. The metagenomes of ayran 13AR and kefir 07KF had a low number of genes encoding GHs, and the most abundant families were GH1 (2–3), GH13 (9–10 genes), GH72 (4–6) and GH73 (5 genes in each) and GH5 (3 genes in each). The common GH families encoded in metagenomes of other products were GH1 (from 20 to 101 genes), GH13 (38–142 genes), GH170 (4–23), GH20 (4–8 genes), GH23 (6–18), GH25 (18–57 genes), GH3 (13–24), GH31 (5–15 genes), GH32 (from 6 to 25 genes), GH43 (5–14), GH5 (2–15), GH65 (from 8 to 25 genes), GH73 (14–60 genes) and GH8 (3–8 genes).

Among GHs were many genes encoding putative beta-galactosidases (enzymes of GH1, GH2, GH35 and GH42 families), required for the hydrolysis of lactose, which is present in milk in a relatively high concentration [74]. Also, plenty of genes encoding the enzymes of the GH13, GH31, GH65 and GH77 families involved in the decomposition of storage carbohydrates (e.g., trehalose or starchlike polysaccharides) were found. Moreover, the genes of peptidoglycan-active enzymes (members of GH20, GH23, GH25, GH73, GH170 families) that could either play role in constructive metabolism or be a part of the defense system against pathogenic microbiota were detected [75]. Some other glycosidases can probably be involved in the degradation of glycoproteins/polysaccharides contained in milk [76,77].

The analysis of glycosyltransferases’ diversity revealed that enzymes of the GT2 (24–253 genes), GT4 (16–143 genes), GT8 (6–24 genes), GT28 (2–22 genes), GT32 (2–22 genes) and GT51 (5–67 genes) families were the most numerous and universal across all the studied samples. GTs are involved in the biosynthesis of exopolysaccharides [78], which influence the texture of products and provide prebiotic, immunomodulatory, antioxidant and other beneficial effects to FMPs [79]. Interestingly, genes encoding some of the minor GT families were present only in part of the FMP metagenomes. Genes of GT15, GT34, GT57, GT62 and GT71 were only found in liquid products and khuruud, but such genes were absent in other cheeses (bryndza, suluguni-like and cottage cheeses). On the other hand, representatives of GT9, GT56 and GT83 were not encoded in the metagenomes of liquid products, excluding khurunga and shubat. Families GT2, GT4, GT8 and GT32 are primarily involved in LPS biosynthesis, biofilm formation and extracellular and capsular polysaccharide biosynthesis [80,81]. GT111 and GT113 were characterized as enzymes involved in galactan biosynthesis [82] and biofilm formation [83]. Members of GT28 and GT51 families are principally involved in the synthesis of the cell wall peptidoglycan and play crucial roles in maintaining the integrity of the cell wall [84]. The enzymes of family GT35 often play a role in storage polysaccharides biosynthesis like starch or glycogen [85].

#### 3.3.2. Secondary Metabolites’ Production

**Figure 4:**
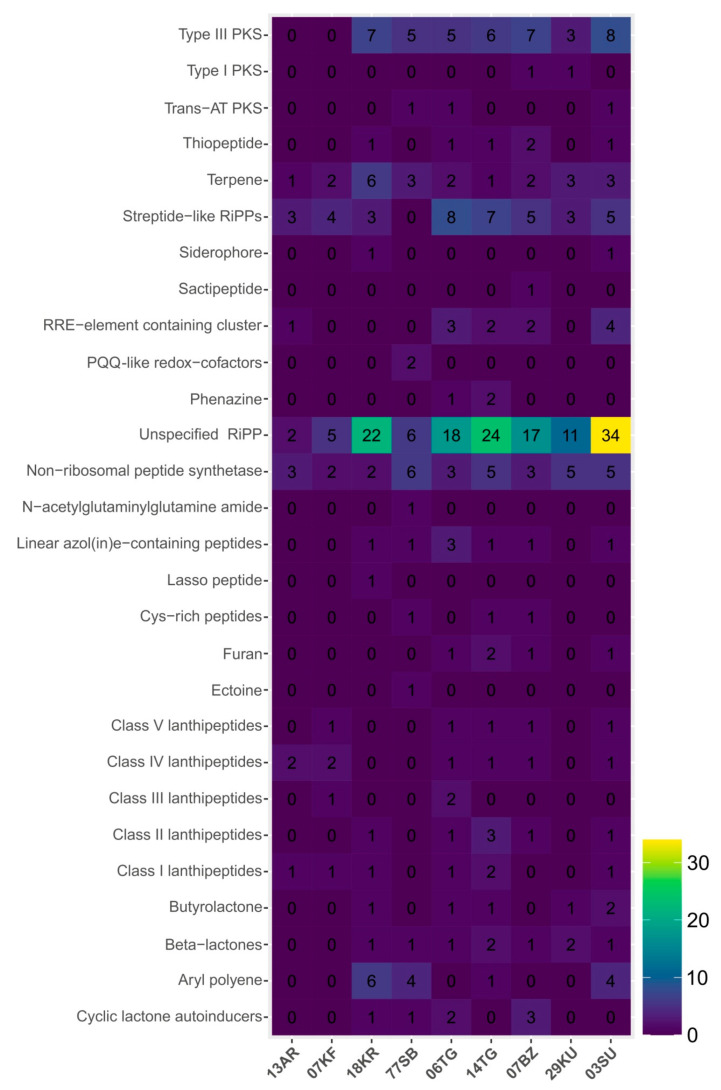
Gene clusters involved in secondary metabolites’ biosynthesis in the metagenomes of the studied fermented milk products.

Bacterial secondary metabolites are widely used as antibiotics, anticancer agents, insecticides and food supplements [86]. The capability of microorganisms to synthesize various secondary metabolites is an important feature. This is especially valid with regard to microorganisms inhabiting food products, FMPs in particular. Secondary metabolites are often synthesized by means of special enzyme machinery, usually encoded by biosynthetic gene clusters [87]. Altogether, 392 clusters involved in the biosynthesis of 28 bioactive compound types were found in the studied metagenomes (Figure 4). The most represented and ubiquitous clusters encoded different types of polyketide synthases (type III PKS, type I PKS and trans-AT PKS), lanthipeptides of classes I-V, other various bacteriocins (RiPP-like and RaS-RiPP), nonribosomal peptide synthetases (NRPS) and enzymes involved in terpens biosynthesis. Some gene clusters characterized by a high total abundance were absent in some metagenomes. PKS gene clusters were absent in the kefir (07KF) and ayran (13AR) metagenomes and lanthipeptides genes in the khuruud (29KU) and shubat (77SB) metagenomes. Also, clusters responsible for the biosynthesis of aryl polyenes (in the 03SU, 14TG, 18KR and 77SB metagenomes), beta-lactones (in the 03SU, 06TG, 07BZ, 14TG, 18KR, 29KU and 77SB metagenomes), butyrolactone (in the 03SU, 06TG, 14TG, 18KR, 29KU metagenomes) and furans (in the 03SU, 06TG, 07BZ and 14TG metagenomes) were identified.

PKS are responsible for multiple condensations of acyl-CoA building blocks resulting in polyketides synthesis. Some polyketides are actively used in medicine, mainly as antibiotics, immunosuppressants, antiparasitic and antitumoral agents, while others are known to be food-spoiling toxins or virulence factors [88]. Nevertheless, T3PKS genes found in significant numbers in the studied metagenomes may indicate the antimicrobial ability of bacteria-inhabited artisanal FMPs against pathogenic bacteria, as it was shown for other LAB [84]. At the same time, we detected the gene clusters of aryl polyenes (a class of PKS products) synthesis in the suluguni-like cheese, cottage cheese, khurunga and shubat metagenomes. For a long time, no aryl polyenes biosynthesis genes had been found in the genomes of lactic acid bacteria [89]. Recently, it was shown that a mammalian gut inhabitant, *Limosilactobacillus reuteri*, also involved in food fermentation [90], produced polyene-like compounds that lysed competitor strains [87]. This may suggest an antimicrobial function of these substances in microbial communities inhabiting the analyzed products. However, a more precise determination of PKS gene clusters’ functions requires more detailed research including in vitro activity detection.

Ribosomally synthesized and post-translationally modified peptides (RiPPs) of bacterial origin (in other words, bacteriocins) are natural compounds that are highly attractive candidates for antibacterial prevention and therapy [91]. Bacteriocins have been used for many years in food preservation and medicine—from their application in active food packaging to their role as antithrombotic, cholesterol-lowering, antihypertensive agents and inhibitors of a wide range of pathogenic and spoilage microorganisms [92]. Most of the secondary metabolite genes identified in the metagenomes of the analyzed artisanal FMPs belonged to unspecified RiPP-like products. This may indicate a potential availability of bacteriocins with unknown features synthesized by bacteria inhabiting these products. The highest number of unspecified RiPPs was found in the metagenome of suluguni-like cheese, where a large proportion of reads were not classified at the species level. Metagenome mining also revealed the presence of all known types of lanthipeptides, including nisin, in almost all artisanal FMPs. Lanthipeptides belong to class I of bacteriocins (small post-translationally modified peptides) and they are active against a large number of Gram-positive pathogens, including antibiotic-resistant strains [93]. The metagenomes of khuruud and shubat samples did not contain lanthipeptide genes; however, the largest number of NRPS genes was found there. Nonribosomally produced peptides, synthetized by NRPS, are mostly cyclic compounds containing nonproteinogenic amino acids, small heterocyclic rings and other unusual modifications in the peptide backbone. A wide spectrum of activities makes them valuable for different applications—from antibiotic precursors or antibiotics themselves (e.g., vancomycin) to immunosuppressive, cytostatic agents or food preservatives [94,95]. The detection of such genes in all metagenomes of the studied FMPs makes these products suitable targets for the isolation and further exploration of substances with high potential for medical and food industries.

Terpenes are a class of hydrocarbons, formally derived from isoprene, produced by representatives of all domains of life, especially by higher plants. They can exhibit various properties such as antimicrobial, antifungal, antiparasitic, antiviral, antiallergenic, anti-inflammatory and chemotherapeutic and are also used as flavoring and aromatic substances in the food and cosmetic industries [96]. Since the ability of LAB to synthesize terpenes is not described thoroughly [97,98], the presence of genes involved in terpenes’ synthesis found in all analyzed metagenomes was intriguing. β-lactones, cyclic carboxylic esters with four-membered rings, have a unique feature in the form of highly reactive electrophilic scaffolds and serve as versatile synthetic intermediates. Many of them described to date possess significant antibacterial, antifungal, anticancer and antiobesity activities [99]. Our metagenome-based analysis revealed genes encoding β-lactones with a potential protease inhibition in the majority of samples. At the same time, we are not aware of any studies on the characterization or detection of β-lactones from lactic acid bacteria. Traditionally, such compounds originated from actinomycetes [99], as well as gamma-butyrolactones [100], for which genes responsible for their synthesis were found by a metagenome analysis of some FMPs studied in this work. As a signaling molecules, γ-butyrolactones are known to be involved in the regulation of differentiation and secondary metabolism in actinomycetes [101]. The role of these compounds in bacteria-inhabited fermented foods is unknown, but it should not be forgotten that in the human body, γ-butyrolactone is converted into γ-hydroxybutyric acid, a naturally occurring neurotransmitter and depressant [102]. That is why it is important to monitor these substances in dairy products, and genomic-based methods can greatly facilitate and simplify this. The detection of furan biosynthesis genes in the metagenomes of exclusively solid products (suluguni-like cheese, cottage cheese, bryndza) most likely indicates that bacteria synthesize such compounds during fermentation and ripening, contributing to the flavor of the product [103], in contrast to fermented milk beverages that are usually odorless.

Thus, numerous gene clusters involved in the biosynthesis of many types of secondary metabolites with potential antimicrobial, immunomodulatory, anticancer effects or an ability to contribute to the organoleptic characteristics were found in the metagenomes of the analyzed artisanal FMPs. However, currently, a precise deciphering of their functions is impossible using only in silico analyses and requires experimental confirmation.

#### 3.3.3. Antibiotic Resistance Systems

Antibiotic resistance represents one of the most serious problems of modern medicine worldwide [104]. Therefore, the detection of the ABR gene clusters in the microbiomes of fermented foods is of undoubted importance for the initial analysis of their beneficial properties. LAB and other microbes from FMPs can possess a natural resistance to a broad spectrum of antibiotics and can be reservoirs of ABR genes [105]. For instance, aminoglycosides, vancomycin and tetracycline resistance genes were detected in LAB stains isolated from dairy beverages and yogurts in China [106]; *Lactobacillus* strains isolated from drink yogurt from Russia and commercial probiotics (Armenia, Bulgary, Russia) were resistant to vancomycin, ciprofloxacin and some aminoglycosides, and the ABR genes were detected in their genomes [107]. A high level of resistance to tetracycline was observed in *Enterococcus faecalis* isolates from Korean fermented soybean foods [108]. Genes responsible for the resistance to tetracycline, macrolides, aminoglycosides, beta-lactams and some other antibiotics were found in the metagenomes of traditional Brazilian cheeses [109]; the gene of multidrug resistance efflux pumps and genes of the resistance to beta-lactams, macrolides, fluoroquinolones, fosfomycin and aminoglycosides were found in Chilean kefirs [45] as well as nunu from Ghana and koumiss from China [73]; and so on. Thereby the presence of ABR genes in the metagenomes of food products should be checked because it can potentially decrease product safety level due to the transfer of ABR genes to pathogens [110]. During the current search of ABR genes in nine metagenomes of FMPs, a total of 121 genes were found (Table S4). The number of ABR systems varied from very low (complete absence in ayran 13AR and the presence of a single gene in kefir (07KF)) to 47 genes in the bryndza sample (07BZ). There were genes responsible for resistance to tetracycline—ribosome protection proteins or efflux pumps (03SU, 06TG, 07BZ, 14TG, 18KR, 29KU), aminoglycosides—exporters and aminoglycoside acetyltransferases, nucleotidyltransferase or phosphotransferase (03SU, 06TG, 07BZ, 14TG, 18KR), cephalosporin—beta-lactamases (06TG, 07BZ, 14TG, 18KR), fluoroquinolones (06TG, 07BZ, 14TG), macrolides—macrolide 2′-phosphotransferase or efflux pumps (07BZ, 07KF, 77SB), peptides—enzymes involved in modifications of surface structures (03SU, 07BZ, 14TG) and some other compounds. Moreover, multiple drug resistance genes were detected in a number of products, mostly in the 07BZ and 14TG samples. It seems that there is a relation between microbial diversity, product texture and ABR: the metagenomes of solid products and products with more complex microbiomes possessed a higher number of ABR genes. Likely, microbial communities with a higher number of species (a) have more antagonistic interactions that cause antibiotic production and consequently increase the requirement of ABR genes and (b) provide an increased probability of ABR genes’ lateral transfer.

## 4. Conclusions

Artisanal FMPs can be a source of still unexplored microbial diversity, and this work contributes to the knowledge of the microbial species inhabiting traditional dairy foods, prepared in different regions of Russia. In the present study, various species of lactic acid bacteria known to be involved in milk fermentation (e.g., *Lb. delbrueckii*, *Lb. helveticus*, *Lentilb. kefiri*, *S. thermophilus*, *Leu. mesenteroides*, *Lc. lactis*, *Lc. cremoris* and others) were found. Moreover, the metagenomic analysis of cottage cheese and shubat allowed us to identify bifidobacteria (*B. mongoliense* and *B. crudilactis*) for the first time in the microbiomes of such product types. Furthermore, unknown *Acetobacter* species were only detected in the metagenomes of shubat and khurunga. In addition, some other species non-canonical for FMPs were found.

The metagenomics of the analyzed FMPs revealed a large diversity of CAZymes, in particular glycosidases involved in lactose hydrolysis during milk fermentation and glycosyltransferases playing a role in exopolysaccharide biosynthesis, which potentially have various beneficial effects such as prebiotic, immunomodulatory and antioxidant effects. Numerous gene clusters of secondary metabolite biosynthesis, including bacteriocins (lanthipeptides and unspecified RiPPs), nonribosomal peptides, polyketides, as well as genes probably involved in the synthesis of nontypical LAB bioactive compounds, such as β-lactones, terpenes and furans, were also found in the analyzed metagenomes. Moreover, the metagenome of suluguni-like cheese possessed the highest number (among the analyzed samples) of unknown bacteriocins, which correlated with a significant proportion of lactic acid bacteria not classified at the species level. All this raises the importance of traditional FMPs not only as a source for new lactic acid bacteria but also as reservoir of novel bioactive molecules with antimicrobial, immunosuppressive and anti-inflammatory activities or bearing attractive flavoring and aromatic properties. The lower representation of antibiotic resistance genes or even their absence in the studied metagenomes also supports the use of such products for new starter cultures’ design or the isolation of new safe probiotics without undesirable antibiotic resistance. Thus, in our case high throughput metagenomic mining provided data useful for the microbial ecology of dairy foods due to its help deciphering the composition of microbiomes, as well as it is necessary for the functional analysis of FMPs, such as discovering new bioactive natural products or the detection of antibiotic resistance genes in genomes of potential probiotics, which is relevant nowadays due to the acquisition or increase in antibiotic resistance of many pathogens.

## Figures and Tables

**Table 1:** Properties of metagenomes of studied fermented milk products.

Sample	Product	Total Bases, Gbp	Assembly Size *, Mbp	Contig Numbers *	N50 *, bp	Coverage *
13AR	Ayran	11.25	14.56	2636	13,759	742
07KF	Kefir	8.8	12.88	11,767	1005	657
18KR	Khurunga	10.27	59.06	24,223	7497	161
77SB	Shubat	7.71	37.05	19,837	6460	178
06TG	Cottage cheese	8.92	40.21	18,001	6553	201
14TG	Cottage cheese	10	52.28	18,472	7639	171
07BZ	Bryndza	7.59	42.50	14,111	18,726	149
29KU	Khuruud	9.58	50.37	33,110	1952	180
03SU	Suluguni-like cheese	8.33	58.66	34,508	2953	115

*—these values were calculated based on contigs with lengths > 500 bp.

## Data Availability

Metagenomic sequences are available in the GenBank database under accession numbers within Bioproject PRJNA907749 (https://www.ncbi.nlm.nih.gov/bioproject/PRJNA907749 accessed on 17 October 2023): JARIFI000000000 (https://www.ncbi.nlm.nih.gov/nuccore/JARIFI000000000 accessed on 17 October 2023), JARIFK000000000 (https://www.ncbi.nlm.nih.gov/nuccore/JARIFK000000000 accessed on 17 October 2023), JARIFL000000000 (https://www.ncbi.nlm.nih.gov/nuccore/JARIFL000000000 accessed on 17 October 2023), JARIFM000000000 (https://www.ncbi.nlm.nih.gov/nuccore/JARIFM000000000 accessed on 17 October 2023), JARIFO000000000 (https://www.ncbi.nlm.nih.gov/nuccore/JARIFO000000000 accessed on 17 October 2023), JARIFQ000000000 (https://www.ncbi.nlm.nih.gov/nuccore/JARIFQ000000000 accessed on 17 October 2023), JARIFR000000000 (https://www.ncbi.nlm.nih.gov/nuccore/JARIFR000000000 accessed on 17 October 2023), JARIFT000000000 (https://www.ncbi.nlm.nih.gov/nuccore/JARIFT000000000 accessed on 17 October 2023), JARIFU000000000 (https://www.ncbi.nlm.nih.gov/nuccore/JARIFU000000000 accessed on 17 October 2023).

## Supplementary Materials

The following supporting information can be downloaded at: https://www.mdpi.com/article/10.3390/microorganisms12010016/s1, Table S1: Relative abundances of different taxa in FMPs metagenome based on read annotation; Table S2: Characteristics of obtained MAGs; Table S3: CAZymes encoded in metagenomes; Table S4: Antibiotic resistance genes found in studied FMPs metagenomes.
